# Cross-reactive inhibitory antibody and memory B cell responses to variant strains of Duffy binding protein II at post-*Plasmodium vivax* infection

**DOI:** 10.1371/journal.pone.0276335

**Published:** 2022-10-18

**Authors:** Pongsakorn Thawornpan, Siriruk Changrob, Piyawan Kochayoo, Kittikorn Wangriatisak, Francis B. Ntumngia, Sai Lata De, Eun-Taek Han, John H. Adams, Patchanee Chootong

**Affiliations:** 1 Department of Clinical Microbiology and Applied Technology, Faculty of Medical Technology, Mahidol University, Bangkok, Thailand; 2 Center for Global Health and Infectious Diseases Research and USF Genomics Program, College of Public Health, University of South Florida, Tampa, Florida, United States of America; 3 Department of Medical Environmental Biology and Tropical Medicine, School of Medicine, Kangwon National University, Chuncheon, Gangwon-do, Republic of Korea; Universidade Federal de Minas Gerais, BRAZIL

## Abstract

Duffy binding protein region II (DBPII) is considered a strong potential vaccine candidate of blood-stage *P*. *vivax*. However, the highly polymorphic nature of this protein often misdirects immune responses, leading them to be strain-specific. Details of cross-reactive humoral immunity to DBPII variants have therefore become an important focus for the development of broadly protective vaccines. Here, cross-reactive humoral immunity against a panel of Thai DBPII variants (DBL-THs) was demonstrated in immunized BALB/c mice and *P*. *vivax* patients, by *in vitro* erythrocyte-binding inhibition assay. Sera from immunized animals showed both strain-transcending (anti-DBL-TH2 and -TH4) and strain-specific (anti-DBL-TH5, -TH6 and -TH9) binding to DBL-TH variants. Using anti-DBL-TH sera at 50% inhibitory concentration (IC50) of the homologous strain, anti-DBL-TH2 sera showed cross inhibition to heterologous DBL-TH strains, whereas anti-DBL-TH5 sera exhibited only strain-specific inhibition. In *P*. *vivax* patients, 6 of 15 subjects produced and maintained cross-reactive anti-DBL-TH inhibitory antibodies through the 1-year post-infection timepoint. Cross-reactive memory B cell (MBC) responses to DBL-TH variants were analyzed in subjects recovered from *P*. *vivax* infection (RC). The plasma samples from 5 RC subjects showed broad inhibition. However, MBC-derived antibodies of these patients did not reveal cross-inhibition. Altogether, broadly anti-DBP variant inhibitory antibodies developed and persisted in *P*. *vivax* infections. However, the presence of cross-reactive anti-DBL-TH inhibitory function post-infection was not related with MBC responses to these variants. More detailed investigation of long-lasting, broadly protective antibodies to DBPII will guide the design of vivax malaria vaccines.

## Introduction

*Plasmodium vivax* is a major causative agent of malaria associated with high morbidity, particularly in tropical and subtropical regions [1]. A rising number of drug-resistant *P*. *vivax* strains, the evolution of more virulent forms of the parasite, as well as the formation of hypnozoites which lead to relapse, necessitate the development of an effective protective vaccine. Among several merozoite proteins involved in *P*. *vivax* invasion, Duffy binding protein (DBP) is a vital microneme protein associated with the decisive, irreversible step of junction formation during the later stages of the invasion process. DBP region II (DBPII) is a ligand domain that binds the Duffy Antigen Receptor for Chemokines (DARC) by dimerization of two DBP and two DARC molecules, forming a heterotetrameric structure [2]. DBPII is a leading *P*. *vivax* vaccine candidate by virtue of its ability to induce inhibitory antibodies that can block DARC-dependent blood-stage invasion [3–6]. Thus, the development of a DBPII-based vaccine has a high potential for protection against or eradication of *P*. *vivax*.

Despite the promising potential of DBP as a vaccine candidate, its naturally occurring polymorphisms often misdirect immune responses so that they become strain-specific, representing a major challenge in vaccine effectiveness [7]. It has been shown that naturally acquired immunity to DBPII is present but weakly effective and short-lived [8]. Reactivity of strain-specific antibody can be altered by even a single amino acid substitution in an allelic variant of DBPII [9]. The target epitopes of anti-DBPII inhibitory antibodies contain most of the prevalent variable residues resulting from DBP allelic diversity [9]. Although strain-transcending inhibitory antibodies are less prevalent, there is a significant correlation of their reactivity to certain epitopes and the inhibition of DBPII-receptor function [10]. This raises the hypothesis that DBP variation is an immune evasion mechanism leading to strain-specific immunity, while broadly neutralizing immunity is achieved when antibodies target functionally-conserved epitopes.

A detailed understanding of cross-reactive immunity to DBPII will enable better vaccine strategies. Studies in endemic regions regularly find a few individual patients who produce high titers of strain-transcending anti-DBPII antibodies [11, 12]. Cross inhibition of heterologous DBPII variants was reported in *P*. *vivax* patients living in low transmission settings [13–17] and in hyperendemic areas [6]. In addition, a novel DBPII vaccine, DEKnull, engineered by extensively altering the polymorphic residues on the DBP ligand domain, more consistently produced a strain-transcending inhibitory response [5, 8, 10, 11]. Besides antibodies, memory B cells (MBCs) against DBPII variants were detected three years post-infection in subjects without re-infection [18]. The details of cross-reactive MBCs in *P*. *vivax*-infected individuals are still lacking, both regarding their development and their persistence [19, 20]. Thus, it is of great importance to interrogate these cross-reactive MBCs which are key to the production of broadly inhibitory antibodies in *P*. *vivax* patients, and better understanding cross-reactive humoral immunity against DBPII variants.

In the present study, we evaluated the cross-reactivity of anti-DBPII variant antibodies in the immunized mice and malaria-infected humans, and examined the persistence of broadly anti-DBPII inhibitory antibodies by monitoring their function following *P*. *vivax* infection, and sought to detect the presence of cross-reactive MBCs. Better understanding the cross reactivity of antibodies and MBCs will help guide development of DBPII-based vaccines against variant vivax malaria in the future.

## Materials and methods

### Ethics statement

This study was approved by the Committee on Human Rights Related to Human Experimentation, Mahidol University, Thailand (MU-IRB 2012/079.240). Written informed consent was obtained from each participant before blood collection. The criteria for enrolment of *P*. *vivax* subjects were as follows: (1) systolic blood pressure higher than 90 mmHg, (2) body temperature lower than 40°C, (3) hematocrit higher than 25%, and (4) age 18 years or above. Subjects who did not meet the criteria were excluded.

Immunization of mice (BALB/c) was performed with approval and strict following of the guidelines of the Institutional Animal Care and Use Committee of the University of South Florida (IACUC protocol IS00006057). Mice were euthanized by CO_2_ asphyxiation followed immediately by exsanguination. Every step in this experiment was cautiously performed to reduce the suffering of tested animals.

### Blood sample preparation

Heparinized blood samples were taken from a malaria low-transmission area in the southern part of Thailand (Rap Ro village, Tha Sae, Chumphon Province). A longitudinal cohort study was initiated with 15 patients followed post-treatment for profiling inhibition patterns of their anti-DBPII antibodies. Subjects whose samples revealed broad inhibition against more than 3 DBPII variants at acute phase (n = 13) were followed up again one year after infection. As one patient was excluded due to the unavailability of a blood sample, convalescence samples from 12 subjects were analyzed at this 1-year time point. A cross-sectional study was performed to assess the presence of cross-reactive MBCs using PBMC samples collected from *P*. *vivax* patients (n = 8) at 1–3 months post-infection. Blood samples from 19 malaria-unexposed persons who lived in Bangkok, Thailand (a non-endemic area) were collected as healthy controls (HCs).

The *P*. *vivax* infection was diagnosed by microscopic examination of both thin and thick blood films, and confirmed by nested PCR. History of previous malaria infections in individual subjects were obtained from the records of the malaria clinic at Vector Borne Disease Unit 11.4.2. Subjects were scheduled for blood sample collection every 3 months for assessment of sub-patent malaria by nested PCR. Staff conducted weekly house to house visits to estimate the incidence of clinical malaria over the study period. Summaries of demographic information of recruited individuals are shown in S1 and S2 Tables.

### DBL-TH recombinant protein expression

The DNA from samples coding for four different Thai DBPII variants (DBL-TH2, -TH4, -TH5 and -TH9) and reference strain Sal I were amplified by PCR (S3 Table). The PCR products were cloned into pET23a+ vectors and expressed following a previous protocol [5]. Briefly, the coding sequences of DBL-TH genes were codon optimized for expression in *E*. *coli*. The resulting plasmids were transformed into *E*. *coli* BL21(DE3) LysE (Invitrogen, Carlsbad, CA, USA). The bacterial cultures were induced by 1.0 mM IPTG for 4 h at 37°C in a shaker flask with shaking at 250 rpm. The inclusion bodies from expressed proteins were solubilized, purified and refolded as previously described [5, 21]. These recombinant proteins were purified by HiTrap SP FF column and ion exchange chromatography (Cytiva, Marlborough, MA, USA) prior to separation by SDS-PAGE.

### Mice immunization by recombinant DBPII variant antigens

Each 6–8 week old female BALB/c mouse was immunized with a single DBPII variant. Before antigen injection, pre-immune serum from each mouse was collected to use as background control for ELISA or EBIA. The mice were randomly assigned into one of five groups (4 mice each) for a prime-boost immunization strategy. The priming dose was 25 μg of one antigen emulsified in TiterMax Gold given subcutaneously. Booster doses were given to mice three weeks later using the same antigen with TiterMax Gold adjuvant in the same amounts. Mice, two weeks after the second immunization, were euthanized before collecting sera by submandibular bleeding. Both pre- and post-immunization sera were stored at -20ºC prior to use.

### Antibody-depletion ELISA

The cross reactivity and/or variant-specific antibodies against different DBL-TH haplotypes were evaluated by antibody depletion ELISA. Briefly, each variant antigen (at 2 μg/mL) was coated on 96-well plates. The wells were blocked with 2.5% BSA in PBS-T and washed by PBS-T before adding mouse serum. Anti-DBL-TH sera were prepared at a dilution of 1:4000 in blocking buffer. Diluted antisera were then depleted by its immunized-variant antigen for 30 min. Subsequently, those antibody-depleted sera were allowed to react with the other heterologous variants by ELISA for evaluation of cross-reactivity among different haplotypes of DBL-TH. The anti-DBL-TH depleted sera that showed no significant difference of inhibitory activity in 3 or more heterologous strains of DBPII compared to the homologous strain were defined as cross-reactive antisera. Experiments were done in duplicate wells and were repeated two times. MSP1-19 was used as unrelated antigen to confirm antibody specificity after depletion. Pre-immune sera were used to determine background reactivity.

### Erythrocyte-binding inhibition assay (EBIA)

All pEGFP plasmids containing genes encoding different DBL-TH haplotypes were constructed by site-directed mutagenesis, following a previous protocol [22]. To assess strain-transcending activity of patient plasma and immunized mouse sera, plasmids encoding DBL-TH variants and DBPII Sal I were transfected into COS-7 cells [18]. Erythrocyte-binding inhibition, after addition of diluted plasma from *P*. *vivax* subjects or undiluted culture supernatant was quantified by counting rosettes in 30 fields of view (magnification, x40). The percent inhibition was calculated using the following equation: % inhibition = [1 - (number of rosettes in the presence of plasma or culture supernatant/number of rosettes in the presence of medium control)] x 100. High inhibition (HI) was considered inhibition greater than or equal to 80% and less than 80% was defined as low inhibition (LI). Experiments were done in triplicate wells and were repeated two times.

### Inhibitory function of anti-DBL-TH sera

To demonstrate the immunogenicity of DBL-TH antigens in inducing cross-reactive antibody responses, anti-DBL-TH2 and -TH5 antisera representing broadly reactive and strain-specific antibodies, respectively, were assayed for *in vitro* binding inhibition. First, 50% inhibition concentrations (IC50) of anti-DBL-TH2 and -TH5 sera were determined by using undiluted and serially diluted antisera (1:1000–1:6000). Then, each antiserum at its IC50 was subsequently used for testing strain-transcending inhibition against a panel of DBL-TH variants by EBIA, as described above. The cross-reactive inhibition was defined when the inhibition of heterologous strain was not significantly different from that of homologous strain. All experiments were performed in triplicate and were independently repeated two times. Mouse anti-DBPII inhibitory monoclonal antibodies (2D10) characterized in a previous study were used as positive control [22] and pre-immune sera served as negative control.

### Breadth of inhibition of *P*. *vivax* patient plasma

EBIA was performed to assess the inhibitory activity of *P*. *vivax* plasma (diluted 1:200) at acute and 1-year post-infection in cohort study and RC subjects in cross-sectional study. Functional inhibition against a panel of DBL-TH antigens was classified as high inhibition (HI) if inhibition was greater than or equal to 80%, or lower inhibition (LI) if the assay result was less than 80%. Samples with HI to more than 3 strains of DBPII were classified as cross-inhibitory plasma. To analyze the inhibition end-point titers to a DBL-TH variant, broadly inhibitory plasma samples (n = 4) were titrated from 1:200 to 1:6400 and tested for inhibition. The end-point titer was defined as the last dilution that had greater than or equal to 80% inhibitory activity. The cross-inhibition between two variants was defined once the difference in end-point titers between those variants was less than or equal to two-fold. Plasma (n = 2) of subjects who displayed high inhibition against DBL-TH variants, as characterized in our previous study [13], were used as positive control, while media only served as negative control.

### Differentiation of MBCs into antibody-secreting cells

Since cross-reactive inhibition against DBL-TH variants was observed at 1-year post-infection, *P*. *vivax* subjects who had recovered from infection for 1–3 months (RCs) and harbored cross-reactive inhibitory antibodies in circulating blood (RC01, RC02, RC03, RC04 and RC06) were used to detect broad MBC responses to DBL-TH variants. PBMCs (1 x 10^6^) from these RC patients (n = 5) and HCs (n = 4) were transferred into 24-well plates containing 1 mL of culture medium (RPMI + 10% FCS) and subsequently differentiated into antibody-secreting cells (ASCs) with stimulation of R848 (2.5 μg/mL; Invivogen, San Diego, CA, USA) and IL-2 (1,000 IU/mL; Peprotech, Rocky Hill, NJ, USA) at 37°C, 5% CO_2_ for 11 days, as previously described [23]. In this culture system memory B cells differentiated into ASCs [24]. Total IgG levels in culture supernatants were measured by human IgG ELISA.

### Enzyme-linked immunosorbent assay (ELISA)

Antibody responses to DBPII antigens were measured by indirect ELISA, as previously described [18]. Briefly, DBL-TH antigens at 2 μg/mL was coated on 96-well plates and held overnight at 4°C. After blocking with 5% skim milk in PBS-T, plates were incubated with mice antisera (before and after depletion), acute-phase plasma (diluted 1:200) of *P*. *vivax*-exposed, or plasma (diluted 1:200) of healthy donors for 1 h at RT. After washing, horseradish peroxidase-conjugated goat anti-human IgG secondary antibodies (Seracare Life Sciences, Milford, MA, USA) at 1:2000 dilution were added into the wells and incubated at RT for 1 hour before addition of TMB substrate. Absorbance was measured at 405 nm. All the samples were assessed in duplicate and mean values were used in the analyses. Acute-phase plasma samples (n = 3) of the vivax malaria patients who showed high OD values to DBL-TH variants, as characterized in previous study [18], were used as positive control and plasma from HCs (n = 3) were used as negative control. Cut-off values were calculated using mean OD values of 15 malaria-naive donors (HCs) plus 2SD.

To detect total IgG levels in culture supernatant, 1μg/ml of anti-human IgG MT91/145 (Mabtech, Nacka Strand, Sweden) was coated on 96-well plates and held overnight at 4°C. After blocking, culture supernatant (undiluted) was added into coated wells followed by detection with horseradish peroxidase-conjugated goat anti-human IgG secondary antibodies. Signal was developed with TMB and read at 405 nm. Plasma samples from *P*. *vivax*-infected patients (n = 2) were used as positive control. Culture supernatant of non-stimulated wells were used as background control.

### Inhibition activity of anti-DBL-TH antibodies in culture supernatants

To assess the inhibitory effect of MBC-secreted immunoglobulins, culture supernatants of 3 patients (RC02, RC03 and RC06), of the 5 whose plasma showed cross inhibition, were chosen as representative of inhibition against 5, 4 and 3 DBL-TH strains, respectively. Undiluted culture supernatants were added into the wells to test inhibition against DBL-TH-2 -TH4, -TH5 and -TH9, and reference strain Sal I, following the above described EBIA protocol. Plasma (n = 2) of RC subjects who displayed high inhibition against DBL-TH variants were used as positive control. Only culture medium was used as negative control.

### ELISPOT

The presence of DBL-TH-specific MBCs was determined with an ELISPOT assay, performed as previously described [18]. In brief, PBMCs from *P*. *vivax*-infected subjects were cultured with R848 and recombinant human IL-2 (Mabtech, Nacka Strand, Sweden) at 37°C in a 5% CO2 incubator for 96 h. Then, the ELISPOT assay was performed by coating with 15 μg/mL of anti-human IgG MT91/145 (Mabtech), 5 μg/mL of DBL-TH and Sal I antigens, or 1 μg/mL of tetanus toxoid (TT) antigen (Merck Millipore, Billerica, MA, USA) onto Multi-Screen IP plates (Merck Millipore). Cells were seeded in duplicate to yield 5 × 10^4^ cells per anti-human IgG-coated well and 1 × 10^6^ cells per antigen-coated well. After overnight incubation, 1 μg/mL of detection antibodies MT78/145 (Mabtech) was added and the mixture incubated for 2 h at RT. After washing, streptavidin-HRP at 1:1000 dilution (Mabtech) was added; plates were incubated for 1 h, then TMB substrate added. Plates were then rinsed with deionized water and analyzed with a Bioreader 5000 ELISPOT Reader. A positive ELISPOT response was defined as detectable spots in duplicate wells with the total spots in the specific antigen-coated wells being at least twice the number of spots detected with the negative control samples. The antigen-uncoated wells were used as negative controls for DBL-TH-specific ASC analysis.

### Statistical analysis

Statistical analysis and figure presentation were performed using GraphPad Prism version 8.4.3 (GraphPad Software, San Diego, CA, USA). One-way analysis of variance and multiple comparison analysis by Bonferroni test were used to analyze and compare the differences in antibody reactivity and percent inhibition. Differences were considered statistically significant when p≤ 0.05.

## Results

### DBL-TH immunization-induced broad antibody responses

**Fig 1:**
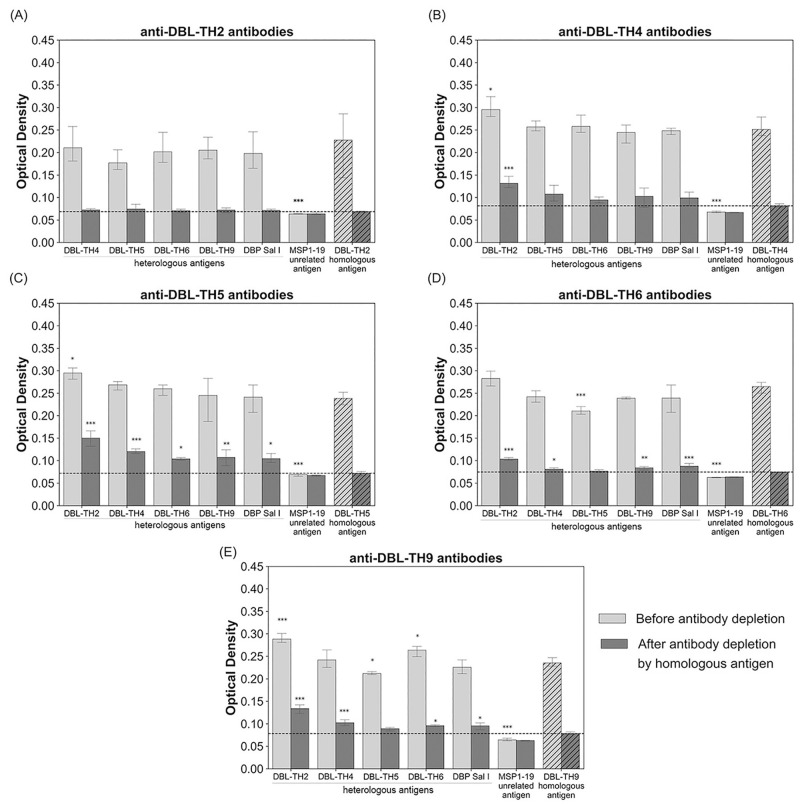
Cross-reactivity of mouse anti-DBL-TH antisera. (A)-(E) Antisera (before and after antibody depletion) against DBL-TH2, -TH4, -TH5, -TH6 and -TH9, respectively, were tested for binding activity against a panel of DBL-TH variants and reference Sal I by ELISA. MSP1-19 protein was used as an unrelated antigen to confirm the specificity of anti-DBL-TH antibody after depletion. The hatched bar represents reactivity against homologous antigen. Dashed line represents the cut-off value for antibody reactivity to homologous strain after antibody depletion. Statistical testing used one-way ANOVA and Bonferroni’s multiple comparison test; * p < 0.05; ** p < 0.01; *** p < 0.001; **** p < 0.0001.

The antisera from mice immunized with DBL-TH2, -TH4, -TH5, -TH6 or -TH9 were used to evaluate the presence of cross-reactive and/or variant-specific antibodies against a panel of DBL-TH variants after antibodies were depleted out by homologous antigen. Compared to homologous strain after complete depletion, the recognition of anti-DBL-TH2 antibodies to each heterologous strain was comparable without differing significantly (Fig 1A). Anti-DBL-TH4 antibodies showed significantly higher reactivity to DBL-TH2 compared to other homologous and heterologous strains (Fig 1B). Anti-DBL-TH5 antibodies exhibited stronger reactivity to all heterologous strains (Fig 1C), while anti-DBL-TH6 and anti-DBL-TH9 antibodies showed significantly higher reactivity to other heterologous strains, except to the DBL-TH5 variant (Fig 1D and 1E). These data indicated that the responses of anti-DBL-TH2 and -TH4 sera were strain-transcending, whereas those of anti-DBL-TH5, -TH6 and -TH9 were strain-specific.

### Anti-DBL-TH antibodies inhibited binding of heterologous antigen to erythrocytes

**Fig 2:**
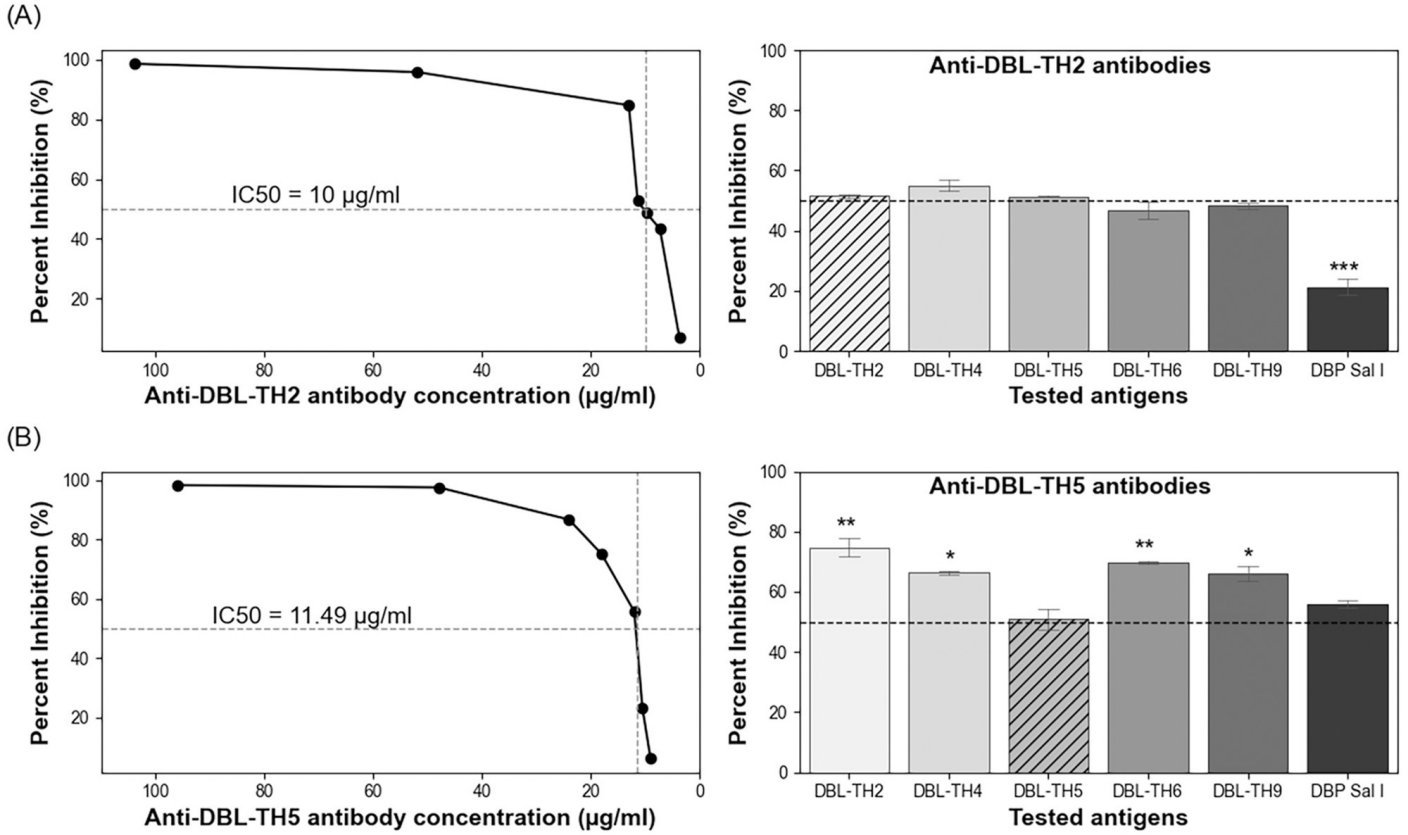
The inhibition of antisera against DBPII variant-erythrocyte binding. (A) The 50% inhibitory concentration (IC50) values and percent inhibition at IC50 of anti-DBL-TH2 antibodies against DBPII reference Sal I and DBL-TH variants (expressed on COS-7 cells). For IC50 determination, each point represents mean percent inhibition of undiluted and diluted antisera (1:1000–1:6000). (B) The 50% inhibitory concentration (IC50) values and percent inhibition at IC50 of anti-DBL-TH5 antibodies against DBPII variants. All inhibition experiments were performed in triplicate and were repeated two times; error bars represent SD. The hatched bar represents percentage inhibition against antigen of homologous strain. Dashed line indicates 50% inhibition. Statistical tests were performed using one-way ANOVA and Bonferroni’s multiple comparison test; * p < 0.05; ** p < 0.01; *** p < 0.001; **** p < 0.0001.

To further analyze the presence of strain-transcending inhibitory antibodies against a panel of DBL-TH antigens, mouse anti-DBL-TH2 and -TH5 antisera (representing broadly reactive and strain-specific responses to DBL-TH variants, respectively) were used to test for inhibitory function. First, IC50 values of anti-DBL-TH2 and -TH5 antisera against erythrocyte binding were determined and found to be 10 μg/ml and 11.49 μg/ml, respectively (Fig 2). Further analysis of inhibitory function of anti-DBL-TH2 antisera against a panel of DBL-TH binding to erythrocytes showed no difference in the inhibitory activity against the DBL-TH variants (except reference Sal I) (Fig 2A). This result indicated that the target epitope of this neutralizing antibody was conserved among this set of Thai variants. For anti-DBL-TH5 antisera, inhibition to a panel of DBL-TH variants (but not to Sal I) differed significantly (Fig 2B), suggesting that there was strain-specificity of anti-DBL-TH5 antibody among the tested Thai variants.

### Cross-inhibition of anti-DBL-TH variants in acute *P*. *vivax* patients

**Fig 3:**
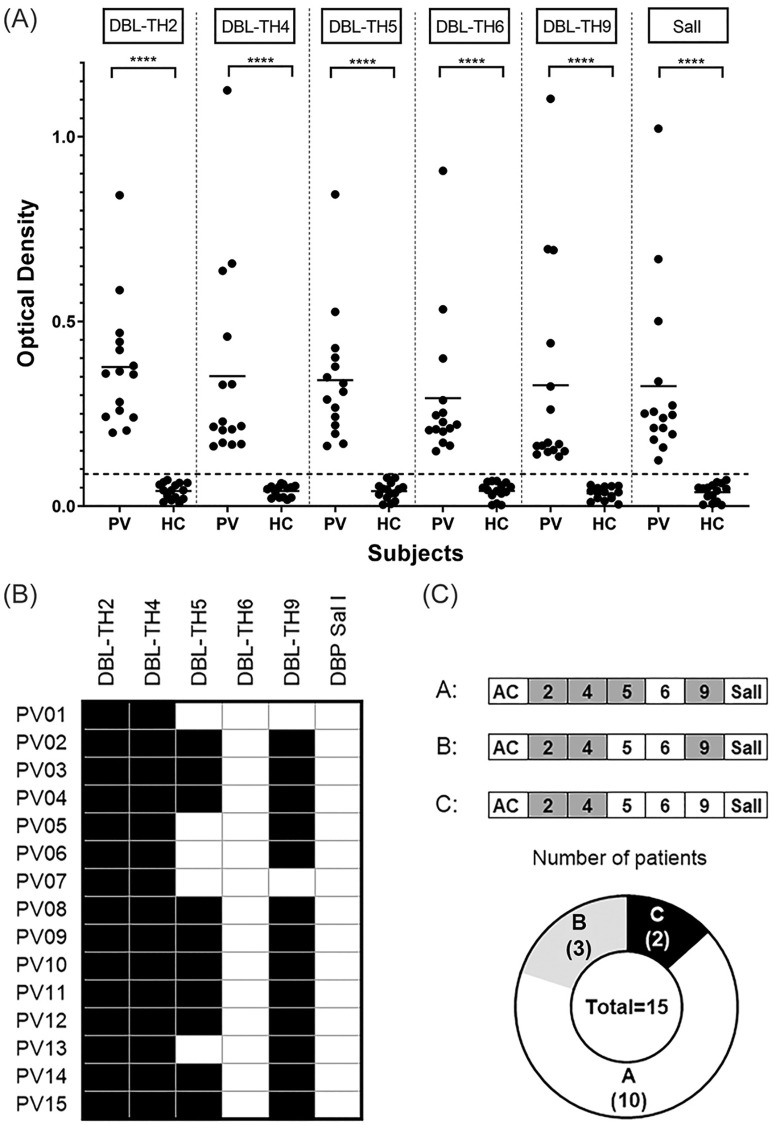
Cross-reactive inhibition profiles of plasma from *P*. *vivax*-infected patients against different DBP-II allelic variants. (A) Serologic responses to DBL-TH variants in acute-phase plasma from 15 *P*. *vivax*-infected subjects (PV) and 15 malaria-naïve donors (HC), detected by ELISA. The dashed line represents the cut-off value calculated from mean of ODs of healthy controls + 2SD. Statistical testing utilized the Mann-Whitney test; **** p < 0.0001. (B) Inhibition patterns of acute-phase plasma from 15 *P*. *vivax*-infected subjects were used to assess inhibition profiles of antibodies against *P*. *vivax* strains with differing DBPII antigens expressed in COS-7 cells. (C) The classification into three groups of inhibitory patterns in acute phase plasma from vivax malaria patients. Black box represents “high inhibition” (HI) defined as inhibition greater than or equal to 80%. White box represents “low inhibition” (LI), inhibition less than 80%.

To assess the persistence of cross-reactive inhibitory antibodies against polymorphic strains of DBL-TH in natural *P*. *vivax* infection, antibody titers and functional inhibition to DBL-TH variants and reference Sal I were tested in plasma collected during acute infection. As expected, all *P*. *vivax*-infected patients had higher antibody reactivity to tested antigens than did healthy controls (Fig 3A). Next, we used plasma of these patients to profile the functional inhibition of anti-DBL-TH antibodies against erythrocyte binding. Plasma from 15 such patients contained antibodies reactive to DBL-TH2, -TH4, -TH5 and -TH9, but none reactive to DBL-TH6 nor reference Sal I (Fig 3B). Based on the inhibition of DBL-TH2, -TH4, -TH5 and -TH9 binding to erythrocytes in individual patients, patterns of cross-strain inhibition were recognized and classified into 3 distinct groups: group A showed cross inhibition against DBL-TH2, -TH4, -TH5 and -TH9; group B showed broad inhibition against DBL-TH2, -TH4 and -TH9; group C exhibited inhibition against DBL-TH2 and -TH4. Plasma from the 15 patients were distributed as 10, 3 and 2 samples in groups A, B and C, respectively (Fig 3C). The samples that showed broad inhibition against more than or equal to 3 DBL-TH variants (group A and B) were selected for subsequent monitoring of cross-reactive responses against DBL-TH antigens in the post-infection period (cohort study).

### Cross reactivity of anti-DBL-TH inhibitory antibodies persisted post-infection

**Fig 4:**
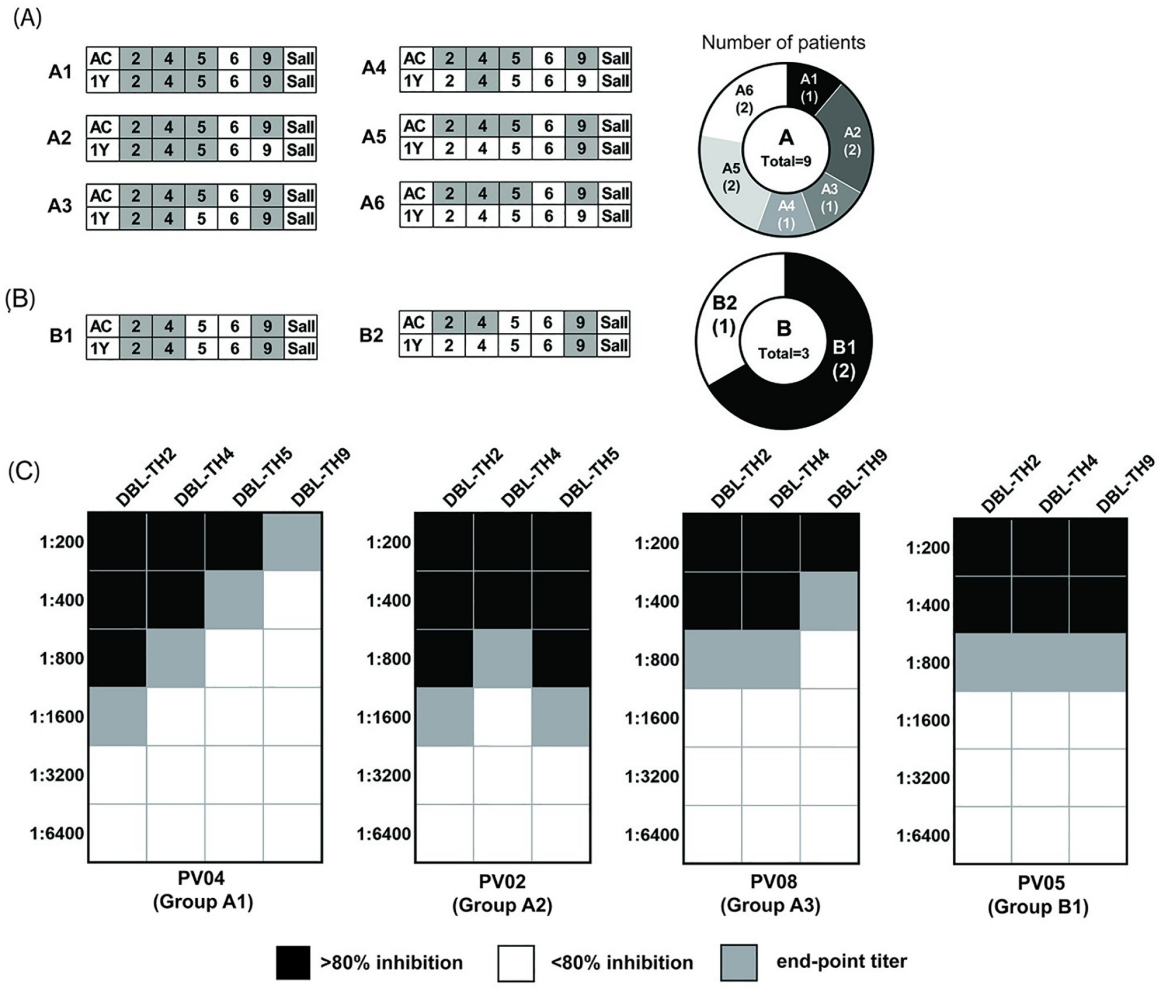
Maintenance of cross-reactive inhibition and end-point titration of plasma at 1-year recovery phase. (A)-(B) Subgroups of inhibition patterns of 12 patients (groups A and B) at acute (AC) and 1-year (1Y) recovery phase with numbers corresponding to patient in parentheses. DBPII variants (DBL-TH2, -TH4, -TH5, -TH6, -TH9 and reference strain Sal I) that could be inhibited were shaded in grey (C) The end-point titration results of four representative patients (A1, A2, A3 and B1) who showed inhibition to more than three variants. The end-point titers are shaded in grey.

The persistence of cross-reactive antibody responses against DBL-TH2, -TH4, -TH5 and -TH9 strains was evaluated in subjects (n = 12) in the cohort study (groups A and B) after they recovered from their *P*. *vivax* infection for one year. From the pattern of inhibition against DBL-TH, there were nine subjects in group A (A1-A6) and three subjects in group B (B1-B2). From group A, 4 samples (A1, n = 1; A2, n = 2; A3, n = 1) maintained cross inhibition against at least three DBL-TH strains. Whereas, 5 patients (A4, n = 1; A5, n = 2; A6, n = 2) showed only short-lived broad inhibition (Fig 4A). In group B, 2 samples (both from B1) showed broad inhibition against DBL-TH2, -TH4 and -TH9, while one sample (from B2) showed inhibition against only DBL-TH 9 (Fig 4B). Collectively, 6 patients maintained broad inhibition 12 months after their episode of vivax malaria, and presented with 3 functional inhibition patterns (A1: DBL-TH2, -TH4, -TH5 and -TH9; A2: DBL-TH2, -TH4 and -TH5; A3/B1: DBL-TH2, -TH4 and -TH9).

Next, to support the persistence of cross inhibition of anti-DBL-TH2, -TH4, -TH5 and TH9 antibodies, plasma samples from subjects with broad inhibition patterns (A1, A2, A3 and B1) were titrated to compare the strength of inhibitory activity. The results clearly revealed that the plasma from A1 (PV04) showed no cross-reactivity to a panel of DBL-TH variants, while plasma from A2 (PV02), A3 (PV08) and B1(PV05) showed broad inhibition. Subject A2 (PV02) showed broad inhibition against DBL-TH2, -TH4 and -TH5, while subjects A3 (PV08) and B1 (PV05) displayed broad inhibition to DBL-TH2, -TH4 and -TH9 (Fig 4C).

### Broadly anti-DBL-TH inhibitory antibodies in patient plasma was not related to those from differentiated MBCs

**Fig 5:**
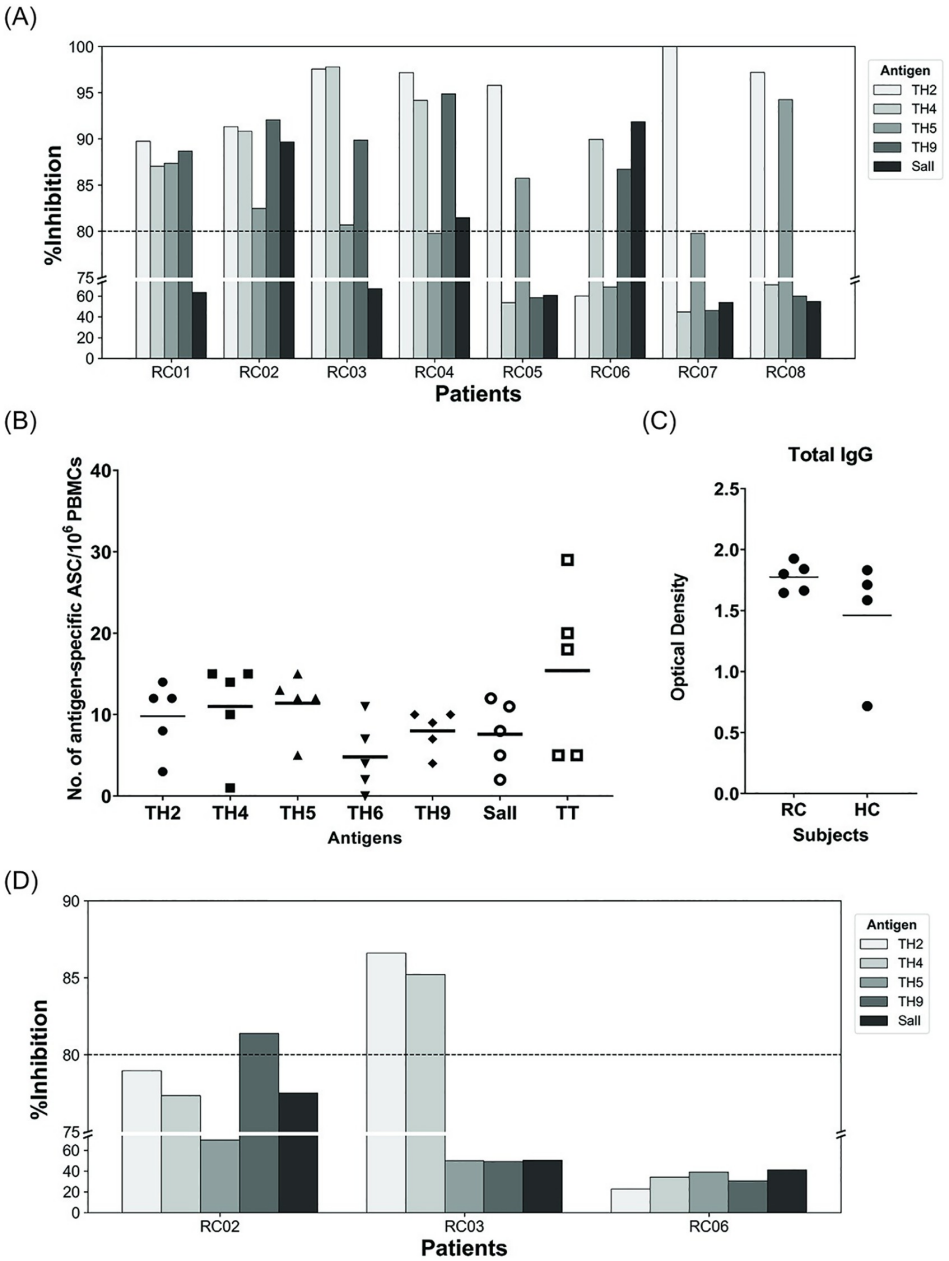
Inhibition patterns of plasma collected at 1–3 months recovery phase and MBC-derived immunoglobulins in culture supernatant assayed against different DBPII haplotypes. (A) Percent inhibition of plasma (diluted 1:200) at recovery phase of 8 patients (RC01-08) against DBL-TH variants and reference strain Sal I. (B) Number of DBL-TH-specific and tetanus toxoid (TT)-specific ASCs per million PBMCs in 5 patients whose plasma showed cross inhibition to more than 3 DBL-TH strains. (C) Optical density (OD) values at 405 nm of total IgG antibodies in culture supernatant from five RC patients (RC) and four healthy controls (HC). (D) Percentage inhibition of undiluted culture supernatant samples of 3 representative RC patients (RC02, RC03 and RC06) whose plasma showed cross-inhibition against 5, 4 and 3 DBL-TH strains, respectively. Statistical tests used were one-way ANOVA and Bonferroni’s multiple comparison test to compare the mean number of spots of DBL-TH-specific ASCs and OD of total IgG. Dashed line in *in vitro* erythrocyte-binding inhibition represents 80% cut-off value for high inhibition (HI).

To explore whether cross-reactive anti-DBL-TH inhibitory antibodies in peripheral blood of RC subjects were related to broad MBC responses, subjects (n = 8) recovered from *P*. *vivax* infection 1–3 months previous were studied, plasma samples from 5 patients showed broad inhibition to a panel of DBL-TH antigens (Fig 5A). As above, the inhibition patterns were classified into one of three groups, including inhibition to 5 strains (RC02), 4 strains (RC01, RC03, RC04) and 3 strains (RC06). Thereafter, PBMCs from these patients (n = 5) were analyzed to detect cross-reactive MBCs to a panel of DBL-TH variants.

MBCs from the 5 patients (RC01, RC02, RC03, RC04 and RC06) were differentiated into ASCs by *in vitro* stimulation cultures. The ASCs were assessed to determine the frequency of DBL-TH-specific ASCs and total IgG levels in culture supernatants. Our results showed that five RC patients harbored MBCs which produced antibodies with specific activity to a panel of DBL-TH variants and the reference strain Sal I (Fig 5B). Moreover, high total IgG responses were detected in supernatants of both RC patients and healthy controls. These data indicated that DBL-TH-specific MBCs were present in RC patients and these differentiated into ASCs after *in vitro* stimulation (Fig 5C). By using EBIA, it was revealed that MBC-derived antibodies from subjects RC02 and RC03 showed inhibition to one strain (DBL-TH-9) and two strains (DBL-TH-2 and DBL-TH4), respectively, while those of RC06 had no inhibitory effects against the set of DBL-TH haplotypes (Fig 5D).

## Discussion

The highly polymorphic nature of DBPII impedes the development of a broadly effective vaccine against *Plasmodium vivax* [25]. Thus, much attention in vaccine development has been given to finding conserved regions within DBPII which function as key components for the induction of strain-transcending antibodies [10, 26, 27]. Here, DBPII variant strains of Thai *P*. *vivax* (DBL-TH) were taken to interrogate the presence of broadly reactive antibodies in immunized mice. In parallel, long-term responses of cross-reactive anti-DBL-TH inhibitory antibodies were assessed in malaria-experienced patients. Few of the immunized mice produced anti-DBL-TH antisera with broad reactivity against DBL-TH variants. The monitoring broadly anti-DBL-TH inhibitory antibodies in *P*. *vivax* patients found that some individuals maintained cross-reactivity after parasite clearance. Although cross-reactive anti-DBL-TH inhibitory antibodies were present in plasma, MBC-derived antibodies from these *P*. *vivax* patients showed no broad inhibition against DBL-TH variants. These data could greatly assist the development and the use of DBPII-based vaccines.

An ultimate goal of an effective PvDBPII-based vaccine is to induce immune responses which inhibit all PvDBPII variants present in malaria-endemic areas [3]. A previous study in a mouse model showed that immunization with combined naturally-occurring DBPII alleles, including P, 7.18 and Sal I, was more immunogenic than with a single allele [5]. Interestingly, antibody responses to a single DBPII-P was strain-specific, while that elicited by mixed alleles was strain-transcending [5]. Another study found that mice developed high antibody responses with a comparable extent of cross-reactivity after immunization with heterologous DBPII variants obtained from Iranian isolates [14]. Here, we performed a mouse immunization study to investigate the immunogenicity of Thai DBPII variants and their capacity to induce cross-reactive anti-DBPII inhibitory responses against erythrocyte binding. After complete depletion of antibodies by homologous antigens, the anti-DBL-TH2 antisera still had broad reactivity against tested antigens and showed strain-transcending inhibition. However, anti-DBL-TH5 antisera exhibited only strain-specific binding and inhibition. Our present findings of cross-reactive inhibitory properties of anti-DBL-TH2 sera suggest the presence of immunodominant conserved epitopes. This would be consistent with a previous study which showed higher immunogenicity of DBL-TH2 compared to other DBL-TH variants [18]. Identification of such dominant conserved epitopes on the DBL-TH2 antigen is important for the development of a DBPII-based vaccine which is protective against diverse *P*. *vivax* infections.

Naturally occurring anti-DBPII inhibitory antibodies that are cross-reactive to DBPII variants are documented in individuals who live in areas with low and seasonal transmission of *P*. *vivax* [13, 14]. A study of malaria-exposed Iranian patients showed cross-reactive antibodies to variant forms of DBPII, contributing to the blocking of erythrocyte invasion via DBPII variants [14]. Likewise, a study in Thai vivax patients demonstrated that some individuals produce strain-transcending antibody responses to DBL-TH variants [13]. Here, persistence of cross-reactive inhibitory antibodies after *P*. *vivax* infection was demonstrated. *P*. *vivax*-infected subjects (PV02, PV05 and PV08) who were followed up for at least 12 months, maintained cross-inhibition to DBL-TH variants but not to the reference Sal I. Remarkably, DBL-TH2 and -TH4 were the most common variants shared in the cross-inhibition profile of studied subjects. This finding indicated that certain *P*. *vivax*-infected individuals were capable of mounting and maintaining inhibitory antibodies specific to multiple Thai DBPII haplotypes widespread in studied endemic area, but not to the reference Salvador 1 strain. Analyses of functional inhibitory antibodies to naturally occurring DBPII alleles are required for best design of efficacious and broadly-protective DBPII-based vaccine strategies. DBP-based vaccines containing multiple DBL-TH2 variants might be essential for protection against the multiple strains of *P*. *vivax* in Thailand and Southeast Asian.

Memory B cells are important for sustaining humoral immunity to malaria [28]. Better understanding of the persistence of protective antibodies and memory B-cells against the diversity of vivax malaria strains can help refine immunization strategies. Information on cross-reactive antibodies has been increasingly accumulating, while that on cross-reactive MBCs has remained quite limited. Here, we addressed the question of whether the maintenance of cross-reactive inhibitory antibodies to DBPII variants is linked to MBC responses against DBL-TH antigens after *P*. *vivax* infection. Our findings revealed that patients who developed strain-transcending inhibitory antibodies did not produce broadly reactive MBC-derived antibodies against DBL-TH variants. Possible explanations of the absence of concordance between circulating cross-reactive inhibitory antibodies and MBC responses to DBP variants include: (i) primary *P*. *vivax* infection triggered cross-reactive naïve B cells to activate and generate ASCs specific to DBL-TH variants with cross-reactive inhibitory anti-DBL-TH antibodies [29]; (ii) triggered B cells produced a low frequency of MBCs cross-reactive against DBL-TH variants [30]; (iii) strain-specific MBCs against dominant DBL-TH variants developed after a primary vivax infection [17]. It has been reported that in dengue virus (DENV) infections significantly increased numbers of cross-reactive DENV-specific plasma cells and MBCs against heterotypic serotypes are detected during the first 2 weeks (post-symptom onset) of secondary DENV infections [31–33]. Thus, the development of cross-reactive humoral immunity may require repeated antigen exposure. To gain better insight into the development of cross-reactive MBCs in response to malaria, further study is needed of these responses in settings where primary and repeated infection can be compared.

Limitations of this study include the relatively small number of patients in the cohort study of long-term broad inhibitory antibodies generated by vivax malaria. The lacking of association between broadly anti-DBL-TH inhibitory antibodies and cross-reactive MBC response to DBL-TH variants could be influenced by the period of *P*. *vivax* infection and the time of sample collection. Since strain-transcending immunity to DBPII develops only after repeated exposure to *P*. *vivax* [34, 35], the patients with re-infections are required to demonstrate the presence of cross-reactive inhibitory antibodies and MBC responses to DBPII variant strains. Further studies using the isolation of DBPII-specific MBCs from *P*. *vivax* patients or immunized animals for the production of monoclonal antibodies are warranted to analyze broad inhibition against a panel of DBPII variants.

In summary, our study demonstrated the presence of broadly inhibitory anti-DBL-TH antibodies in both mice immunized by DBL-TH variants and *P*. *vivax*-infected human. Some patients maintained cross-reactive inhibitory antibodies through the convalescence phase for at least a year, whereas MBC-derived IgG exhibited only a limited breadth of inhibition towards DBL-TH variants. The insights into cross-reactive immunity will allow improvements in the design strategies of conserved epitope-targeting vaccines that are protective against diverse strains of *P*. *vivax* in the future.

## Supporting information

S1 Table Characteristics of acute, recovered *P*. *vivax* patients and healthy subjects recruited for the assessment of antibody responses to DBP variants. (DOCX)

S2 Table Characteristics of recovered *P*. *vivax* patients and healthy subjects recruited for the assessment of cross-reactivity to DBP variants, using 11-day culture supernatant. (DOCX)

S3 Table Panel of DBL-TH alleles used for protein expressions. Polymorphic residues within DBPII and positions with reference to DBPII-Sal I (bold) are indicated. Conserved residues are represented by a dot (.).
(DOCX)

S1 File Raw data for Figs 1–5. (DOCX)
