# Correction: Cross-reactive inhibitory antibody and memory B cell responses to variant strains of Duffy binding protein II at post-*Plasmodium vivax* infection

**DOI:** 10.1371/journal.pone.0320801

**Published:** 2025-03-17

**Authors:** Pongsakorn Thawornpan, Siriruk Changrob, Piyawan Kochayoo, Kittikorn Wangriatisak, Francis B. Ntumngia, Sai Lata De, Eun-Taek Han, John H. Adams, Patchanee Chootong

In the Ethics statement subsection of the Materials and methods, there is an error in the first sentence of the first paragraph. The correct sentence is: This study was approved by the Committee on Human Rights Related to Human Experimentation, Mahidol University, Thailand (MU-IRB 2012/079.2408).
