# Supplementary material for: Cross-reactive inhibitory antibody and memory B cell responses to variant strains of Duffy binding protein II at post-Plasmodium vivax infection
Source: PLoS One. 2022 Oct 18;17(10):e0276335. doi: 10.1371/journal.pone.0276335 (PMC9578595; doi:10.1371/journal.pone.0276335)
Supplement: S1 Table — (DOCX) [file pone.0276335.s001.docx]

**S1 Table.** Characteristics of acute, recovered *P. vivax* patients and healthy subjects recruited for the assessment of antibody responses to DBP variants**.**

| **Characteristics** | **Acute *P. vivax* patients** | **Recovered *P. vivax* patients  (12 months)** | **Healthy subjects** |
| --- | --- | --- | --- |
| Total Number | 15 | 15 | 15 |
| **Age (years)** | | | |
| Median (Q1, Q3) | 35.0 (30.5, 48.5) | 35.0 (30.5, 48.5) | 25.0 (24.0, 28.5) |
| **Gender** | | | |
| Male | 60% (9/15) | 60% (9/15) | 33.33% (5/15) |
| Female | 40% (6/15) | 40% (6/15) | 66.67% (10/15) |
| **Nationality** | | | |
| Thai | 80% (12/15) | 80% (12/15) | 100% (15/15) |
| Myanmar | 20% (3/15) | 20% (3/15) | 0% (0/15) |
| **No. of prior infection** | | | |
| 0 | 15 | 14 | 15 |
| 1 | 0 | 1 | 0 |
| >1 | 0 | 0 | 0 |
| No. of recorded  re-infections | 0 | 1 | 0 |
| Parasitemia (parasite/µL)  Mean ± SD (range) | 6379.56 ± 4697.37  (500-13,000) | 0 | 0 |
