# Supplementary material for: Cross-reactive inhibitory antibody and memory B cell responses to variant strains of Duffy binding protein II at post-Plasmodium vivax infection
Source: PLoS One. 2022 Oct 18;17(10):e0276335. doi: 10.1371/journal.pone.0276335 (PMC9578595; doi:10.1371/journal.pone.0276335)
Supplement: S2 Table — (DOCX) [file pone.0276335.s002.docx]

**S2 Table.** Characteristics of recovered *P. vivax* patients and healthy subjects recruited for the assessment of cross-reactivity to DBP variants, using 11-day culture supernatant.

| **Characteristics** | **Recovered *P. vivax* patients (1-3 months)** | **Healthy subjects** |
| --- | --- | --- |
| Total Number | 8 | 4 |
| **Age (years)** | | |
| Median (Q1, Q3) | 37.5 (23.0, 42.5) | 24.5 (23.75, 25.25) |
| **Gender** | | |
| Male | 62.5% (5/8) | 25% (1/4) |
| Female | 37.5% (3/8) | 75% (3/4) |
| **Nationality** | | |
| Thai | 8 | 4 |
| **No. of prior infection** | | |
| 0 | 8 | 0 |
| 1 | 0 | 0 |
| >1 | 0 | 0 |
| No. of recorded re-infections | 0 | 0 |
| Parasitemia (parasite/µL) | 0 | 0 |
