# Supplementary material for: Cross-reactive inhibitory antibody and memory B cell responses to variant strains of Duffy binding protein II at post-Plasmodium vivax infection
Source: PLoS One. 2022 Oct 18;17(10):e0276335. doi: 10.1371/journal.pone.0276335 (PMC9578595; doi:10.1371/journal.pone.0276335)
Supplement: S3 Table — Polymorphic residues within DBPII and positions with reference to DBPII-Sal I (bold) are indicated. Conserved residues are represented by a dot (.). (DOCX) [file pone.0276335.s003.docx]

**S3 Table. Panel of DBL-TH alleles used for protein expressions.** Polymorphic residues within DBPII and positions with reference to DBPII-Sal I (bold) are indicated. Conserved residues are represented by a dot (.).

| **DBPII alleles** | **Amino acid position** | | | | | | | | | | | | | | |
| --- | --- | --- | --- | --- | --- | --- | --- | --- | --- | --- | --- | --- | --- | --- | --- |
|  | **308** | **313** | **333** | **371** | **375** | **384** | **385** | **386** | **390** | **417** | **424** | **433** | **437** | **475** | **503** |
| DBPII-Sal I | R | . | L | K | . | D | E | K | R | N | L | . | W | P | I |
| DBL-TH2 | . | . | F | E | . | G | K | Q | . | K | I | . | R | . | . |
| DBL-TH4 | . | . | F | . | . | G | K | Q | H | K | I | . | R | . | K |
| DBL-TH5 | . | . | . | E | . | G | . | N | . | K | I | . | R | . | K |
| DBL-TH6 | . | . | . | . | . | G | . | H | . | . | . | . | . | . | . |
| DBL-TH9 | . | . | F | . | . | . | . | . | . | . | I | . | R | . | K |
