# Supplementary material for: Cross-reactive inhibitory antibody and memory B cell responses to variant strains of Duffy binding protein II at post-Plasmodium vivax infection
Source: PLoS One. 2022 Oct 18;17(10):e0276335. doi: 10.1371/journal.pone.0276335 (PMC9578595; doi:10.1371/journal.pone.0276335)
Supplement: S1 File — (DOCX) [file pone.0276335.s004.docx]

**S4 File. Minimal Dataset Manuscript ID PONE-D-22-15715, titled "Cross-reactive inhibitory antibody and memory B cell responses to variant strains of Duffy Binding Protein II at post-*Plasmodium vivax* infection”**

**Fig 1A**

|  | **Before depletion** | | **After depletion** | |
| --- | --- | --- | --- | --- |
|  | **Mean** | **SD** | **Mean** | **SD** |
| **DBL-TH2** | 0.22767 | 0.07431 | 0.06833 | 0.00153 |
| **DBL-TH4** | 0.21033 | 0.04165 | 0.07233 | 0.00252 |
| **DBL-TH5** | 0.17700 | 0.02512 | 0.07433 | 0.00929 |
| **DBL-TH6** | 0.20133 | 0.03785 | 0.07067 | 0.00416 |
| **DBL-TH9** | 0.20533 | 0.02532 | 0.07200 | 0.00557 |
| **DBP-SalI** | 0.19800 | 0.04253 | 0.07133 | 0.00231 |
| **MSP1-19** | 0.06333 | 0.00058 | 0.06367 | 0.00208 |

**Fig 1B**

|  | **Before depletion** | | **After depletion** | |
| --- | --- | --- | --- | --- |
|  | **Mean** | **SD** | **Mean** | **SD** |
| **DBL-TH2** | 0.29533 | 0.02485 | 0.13133 | 0.01365 |
| **DBL-TH4** | 0.25133 | 0.02397 | 0.08133 | 0.00416 |
| **DBL-TH5** | 0.25667 | 0.01172 | 0.10733 | 0.01790 |
| **DBL-TH6** | 0.25833 | 0.02139 | 0.09433 | 0.00651 |
| **DBL-TH9** | 0.24467 | 0.02098 | 0.10267 | 0.02219 |
| **DBP-SalI** | 0.24833 | 0.00737 | 0.09900 | 0.01539 |
| **MSP1-19** | 0.06767 | 0.00208 | 0.06667 | 0.00058 |

**Fig 1C**

|  | **Before depletion** | | **After depletion** | |
| --- | --- | --- | --- | --- |
|  | **Mean** | **SD** | **Mean** | **SD** |
| **DBL-TH2** | 0.29467 | 0.01266 | 0.15000 | 0.01709 |
| **DBL-TH4** | 0.26833 | 0.01002 | 0.12000 | 0.00529 |
| **DBL-TH5** | 0.23833 | 0.01401 | 0.07167 | 0.00513 |
| **DBL-TH6** | 0.25967 | 0.01274 | 0.10367 | 0.00473 |
| **DBL-TH9** | 0.24500 | 0.05103 | 0.10733 | 0.01756 |
| **DBP-SalI** | 0.24100 | 0.03110 | 0.10433 | 0.01041 |
| **MSP1-19** | 0.06900 | 0.00346 | 0.06700 | 0.00173 |

**Fig 1D**

|  | **Before depletion** | | **After depletion** | |
| --- | --- | --- | --- | --- |
|  | **Mean** | **SD** | **Mean** | **SD** |
| **DBL-TH2** | 0.28267 | 0.01650 | 0.10333 | 0.00351 |
| **DBL-TH4** | 0.24167 | 0.01258 | 0.08100 | 0.00361 |
| **DBL-TH5** | 0.21033 | 0.00874 | 0.07667 | 0.00351 |
| **DBL-TH6** | 0.26467 | 0.01286 | 0.07467 | 0.00058 |
| **DBL-TH9** | 0.23867 | 0.00208 | 0.08400 | 0.00436 |
| **DBP-SalI** | 0.23900 | 0.03061 | 0.08767 | 0.00551 |
| **MSP1-19** | 0.06267 | 0.00058 | 0.06367 | 0.00058 |

**Fig 1E**

|  | **Before depletion** | | **After depletion** | |
| --- | --- | --- | --- | --- |
|  | **Mean** | **SD** | **Mean** | **SD** |
| **DBL-TH2** | 0.28833 | 0.01102 | 0.13400 | 0.00985 |
| **DBL-TH4** | 0.24200 | 0.01997 | 0.10233 | 0.00651 |
| **DBL-TH5** | 0.21167 | 0.00404 | 0.08900 | 0.00436 |
| **DBL-TH6** | 0.26367 | 0.01274 | 0.09567 | 0.00208 |
| **DBL-TH9** | 0.23533 | 0.01021 | 0.07833 | 0.00321 |
| **DBP-SalI** | 0.22533 | 0.01563 | 0.09533 | 0.00764 |
| **MSP1-19** | 0.06400 | 0.00346 | 0.06267 | 0.00058 |

**Fig 2A**

| **Anti-DBL-TH2  antibody  concentration (µg/ml)** | **mean of percent  inhibition** | **SD** |
| --- | --- | --- |
| 104 | 98.61 | 0.00826 |
| 52 | 95.83 | 0.00337 |
| 26 | 93.06 | 0.00455 |
| 19.5 | 91.67 | 0.00707 |
| 13 | 84.72 | 0.00659 |
| 11.38 | 52.78 | 0.00799 |
| 9.75 | 48.61 | 0.00955 |
| 7.3 | 43.43 | 0.00114 |
| 3.7 | 7.07 | 0.00360 |

|  | **Percent inhibition** | |
| --- | --- | --- |
| **antigen** | **mean** | **SD** |
| DBL-TH2 | 51.51 | 0.933381 |
| DBL-TH4 | 55.02 | 2.672864 |
| DBL-TH5 | 51.39 | 0.162635 |
| DBL-TH6 | 46.91 | 4.023438 |
| DBL-TH9 | 48.22 | 1.202082 |
| DBP Sal I | 21.33 | 3.655742 |

**Fig 2B**

| **Anti-DBL-TH5  antibody  concentration (µg/ml)** | **mean of percent  inhibition** | **SD** |
| --- | --- | --- |
| 96 | 98.45 | 0.006731 |
| 48 | 97.67 | 0.000984 |
| 24 | 86.82 | 0.001504 |
| 18 | 75.19 | 0.007285 |
| 12 | 55.81 | 0.004052 |
| 10.5 | 23.26 | 0.001097 |
| 9 | 6.2 | 0.007952 |

|  | **Percent inhibition** | |
| --- | --- | --- |
| **antigen** | **mean** | **SD** |
| DBL-TH2 | 74.8000 | 4.5113 |
| DBL-TH4 | 66.3250 | 0.9829 |
| DBL-TH5 | 69.8300 | 4.5285 |
| DBL-TH6 | 66.1700 | 0.5091 |
| DBL-TH9 | 55.9950 | 3.5780 |
| DBP Sal I | 50.9650 | 1.5344 |

**Fig 3A**

|  | **DBL-TH2** | | **DBL-TH4** | | **DBL-TH5** | | **DBL-TH6** | | **DBL-TH9** | | **Sal I** | |
| --- | --- | --- | --- | --- | --- | --- | --- | --- | --- | --- | --- | --- |
| **Patient ID** | **PV** | **HC** | **PV** | **HC** | **PV** | **HC** | **PV** | **HC** | **PV** | **HC** | **PV** | **HC** |
| PV01 | 0.365 | 0.009 | 0.162 | 0.017 | 0.169 | 0.004 | 0.149 | 0.003 | 0.147 | 0.005 | 0.124 | 0.071 |
| PV02 | 0.469 | 0.011 | 0.459 | 0.021 | 0.428 | 0.006 | 0.287 | 0.003 | 0.441 | 0.011 | 0.338 | 0.003 |
| PV03 | 0.357 | 0.016 | 0.215 | 0.023 | 0.31 | 0.013 | 0.202 | 0.007 | 0.172 | 0.014 | 0.195 | 0.004 |
| PV04 | 0.282 | 0.02 | 0.172 | 0.023 | 0.196 | 0.023 | 0.172 | 0.03 | 0.149 | 0.022 | 0.18 | 0.006 |
| PV05 | 0.585 | 0.024 | 0.657 | 0.024 | 0.526 | 0.035 | 0.533 | 0.034 | 0.693 | 0.022 | 0.669 | 0.012 |
| PV06 | 0.38 | 0.028 | 0.217 | 0.038 | 0.349 | 0.046 | 0.246 | 0.039 | 0.168 | 0.027 | 0.256 | 0.027 |
| PV07 | 0.242 | 0.043 | 0.167 | 0.041 | 0.163 | 0.047 | 0.164 | 0.041 | 0.134 | 0.036 | 0.159 | 0.028 |
| PV08 | 0.359 | 0.043 | 0.208 | 0.046 | 0.289 | 0.051 | 0.221 | 0.044 | 0.164 | 0.038 | 0.247 | 0.042 |
| PV09 | 0.423 | 0.043 | 0.637 | 0.049 | 0.402 | 0.054 | 0.4 | 0.05 | 0.696 | 0.038 | 0.501 | 0.047 |
| PV10 | 0.205 | 0.056 | 0.229 | 0.051 | 0.333 | 0.063 | 0.211 | 0.052 | 0.152 | 0.041 | 0.212 | 0.048 |
| PV11 | 0.842 | 0.058 | 1.126 | 0.053 | 0.844 | 0.077 | 0.908 | 0.056 | 1.103 | 0.049 | 0.251 | 0.05 |
| PV12 | 0.24 | 0.063 | 0.329 | 0.053 | 0.219 | 0.077 | 0.228 | 0.064 | 0.324 | 0.053 | 1.022 | 0.05 |
| PV13 | 0.199 | 0.063 | 0.168 | 0.055 | 0.242 | 0.032 | 0.206 | 0.066 | 0.14 | 0.054 | 0.239 | 0.056 |
| PV14 | 0.445 | 0.065 | 0.33 | 0.06 | 0.378 | 0.034 | 0.253 | 0.068 | 0.262 | 0.055 | 0.273 | 0.062 |
| PV15 | 0.259 | 0.071 | 0.206 | 0.062 | 0.267 | 0.045 | 0.208 | 0.068 | 0.163 | 0.057 | 0.212 | 0.065 |
| **mean** | **0.3768** | **0.040867** | **0.352133** | **0.041067** | **0.341** | **0.040467** | **0.292533** | **0.041667** | **0.3272** | **0.0348** | **0.3252** | **0.038067** |

**Fig 3B and 3C**

| **Patient ID** | **Percent inhibition against DBP haplotypes** | | | | | |
| --- | --- | --- | --- | --- | --- | --- |
|  |  |  |  |  |  |  |
|  | **DBL-TH2** | **DBL-TH4** | **DBL-TH5** | **DBL-TH6** | **DBL-TH9** | **DBP Sal I** |
| PV01 | >80% | >80% | <80% | <80% | <80% | <80% |
| PV02 | >80% | >80% | >80% | <80% | >80% | <80% |
| PV03 | >80% | >80% | >80% | <80% | >80% | <80% |
| PV04 | >80% | >80% | >80% | <80% | >80% | <80% |
| PV05 | >80% | >80% | <80% | <80% | >80% | <80% |
| PV06 | >80% | >80% | <80% | <80% | >80% | <80% |
| PV07 | >80% | >80% | <80% | <80% | <80% | <80% |
| PV08 | >80% | >80% | >80% | <80% | >80% | <80% |
| PV09 | >80% | >80% | >80% | <80% | >80% | <80% |
| PV10 | >80% | >80% | >80% | <80% | >80% | <80% |
| PV11 | >80% | >80% | >80% | <80% | >80% | <80% |
| PV12 | >80% | >80% | >80% | <80% | >80% | <80% |
| PV13 | >80% | >80% | <80% | <80% | >80% | <80% |
| PV14 | >80% | >80% | >80% | <80% | >80% | <80% |
| PV15 | >80% | >80% | >80% | <80% | >80% | <80% |

**Fig 4A&4B**

| **Subjects** | **Phase** | **DBL-TH2** | **DBL-TH4** | **DBL-TH5** | **DBL-TH6** | **DBL-TH9** | **DBP-Sal I** | **Pattern** |
| --- | --- | --- | --- | --- | --- | --- | --- | --- |
| PV04 | AC | >80% | >80% | >80% | <80% | >80% | <80% | A1 |
|  | 1yr | >80% | >80% | >80% | ND | >80% | ND |  |
| PV02, PV03 | AC | >80% | >80% | >80% | <80% | >80% | <80% | A2 |
|  | 1yr | >80% | >80% | >80% | ND | <80% | ND |  |
| PV08 | AC | >80% | >80% | >80% | <80% | >80% | <80% | A3 |
|  | 1yr | >80% | >80% | <80% | ND | >80% | ND |  |
| PV10 | AC | >80% | >80% | >80% | <80% | >80% | <80% | A4 |
|  | 1yr | <80% | >80% | <80% | ND | <80% | ND |  |
| PV14, PV15 | AC | >80% | >80% | >80% | <80% | >80% | <80% | A5 |
|  | 1yr | <80% | <80% | <80% | ND | >80% | ND |  |
| PV11, PV12 | AC | >80% | >80% | >80% | <80% | >80% | <80% | A6 |
|  | 1yr | <80% | <80% | <80% | ND | <80% | ND |  |
| PV05, PV06 | AC | >80% | >80% | <80% | <80% | >80% | <80% | B1 |
|  | 1yr | >80% | >80% | ND | ND | >80% | ND |  |
| PV13 | AC | >80% | >80% | <80% | <80% | >80% | <80% | B2 |
|  | 1yr | <80% | <80% | <80% | ND | >80% | ND |  |
| PV09 | AC | >80% | >80% | >80% | <80% | >80% | <80% | - |
|  | 1yr | NA | NA | NA | ND | NA | ND |  |

**Fig4C**

| **Mean percent inhibition for PV02** | | | |
| --- | --- | --- | --- |
|  | DBL-TH2 | DBL-TH4 | DBL-TH5 |
| 1:200 | 98.95 | 98.43 | 98.95 |
| 1:400 | 97.91 | 83.25 | 92.67 |
| 1:800 | 91.62 | 79.58 | 91.1 |
| 1:1600 | 87.96 | 59.16 | 85.34 |
| 1:3200 | 68.59 | 51.83 | 73.3 |
| 1:6400 | 59.16 | 32.98 | 61.78 |

| **Mean percent inhibition for PV04** | | | | |
| --- | --- | --- | --- | --- |
|  | DBL-TH2 | DBL-TH4 | DBL-TH5 | DBL-TH9 |
| 1:200 | 98.95 | 98.95 | 98.95 | 89.53 |
| 1:400 | 98.95 | 95.81 | 93.72 | 60.73 |
| 1:800 | 96.34 | 84.29 | 76.44 | 53.4 |
| 1:1600 | 83.25 | 69.11 | 54.97 | 42.41 |
| 1:3200 | 70.68 | 65.97 | 42.93 | 36.65 |
| 1:6400 | 55.5 | 63.35 | 34.55 | 31.94 |

| **Mean percent inhibition for PV05** | | | |
| --- | --- | --- | --- |
|  | DBL-TH2 | DBL-TH4 | DBL-TH9 |
| 1:200 | 99.48 | 99.48 | 99.48 |
| 1:400 | 99.48 | 96.34 | 98.43 |
| 1:800 | 85.34 | 87.43 | 81.68 |
| 1:1600 | 64.92 | 69.63 | 73.82 |
| 1:3200 | 42.41 | 42.41 | 36.65 |
| 1:6400 | 28.27 | 26.7 | 31.41 |

**Fig5A**

| **Subject** | **Antigen** | **Mean of  percent inhibition** |
| --- | --- | --- |
| RC01 | TH2 | 89.74 |
| RC01 | TH4 | 87.03 |
| RC01 | TH5 | 87.36 |
| RC01 | TH9 | 88.65 |
| RC01 | SalI | 63.23 |
| RC02 | TH2 | 91.31 |
| RC02 | TH4 | 90.82 |
| RC02 | TH5 | 82.46 |
| RC02 | TH9 | 92.04 |
| RC02 | SalI | 89.66 |
| RC03 | TH2 | 97.54 |
| RC03 | TH4 | 97.79 |
| RC03 | TH5 | 80.69 |
| RC03 | TH9 | 89.86 |
| RC03 | SalI | 66.85 |
| RC04 | TH2 | 97.16 |
| RC04 | TH4 | 94.16 |
| RC04 | TH5 | 79.77 |
| RC04 | TH9 | 94.86 |
| RC04 | SalI | 81.48 |
| RC05 | TH2 | 95.78 |
| RC05 | TH4 | 53.46 |
| RC05 | TH5 | 85.75 |
| RC05 | TH9 | 58.46 |
| RC05 | SalI | 60.73 |
| RC06 | TH2 | 59.94 |
| RC06 | TH4 | 89.94 |
| RC06 | TH5 | 68.51 |
| RC06 | TH9 | 86.71 |
| RC06 | SalI | 91.85 |
| RC07 | TH2 | 100 |
| RC07 | TH4 | 44.66 |
| RC07 | TH5 | 79.77 |
| RC07 | TH9 | 45.91 |
| RC07 | SalI | 53.67 |
| RC08 | TH2 | 97.19 |
| RC08 | TH4 | 70.57 |
| RC08 | TH5 | 94.25 |
| RC08 | TH9 | 59.89 |
| RC08 | SalI | 54.64 |

**Fig5B**

|  | **Number of antigen-specific ASC/10^6^ PBMCs** | | | | | | |
| --- | --- | --- | --- | --- | --- | --- | --- |
| **Subjects** | **TH2** | **TH4** | **TH5** | **TH6** | **TH9** | **SalI** | **TT** |
| RC01 | 12 | 10 | 12 | 11 | 10 | 5 | 29 |
| RC02 | 12 | 15 | 12 | 7 | 7 | 8 | 18 |
| RC03 | 14 | 14 | 13 | 0 | 10 | 12 | 20 |
| RC04 | 8 | 15 | 15 | 4 | 9 | 11 | 5 |
| RC06 | 3 | 1 | 5 | 2 | 4 | 2 | 5 |
| **mean** | **9.8** | **11** | **11.4** | **4.8** | **8** | **7.6** | **15.4** |

**Fig5C**

| **Optical density (OD) values at 405 nm of total IgG antibodies in culture supernatant** | | | | | | | | | | |
| --- | --- | --- | --- | --- | --- | --- | --- | --- | --- | --- |
| **RC** | **HC** |  |  |  |  |  |  |  |  |  |
| 1.841 | 1.831 |  |  |  |  |  |  |  |  |  |
| 1.925 | 1.711 |  |  |  |  |  |  |  |  |  |
| 1.664 | 0.717 |  |  |  |  |  |  |  |  |  |
| 1.800 | 1.585 |  |  |  |  |  |  |  |  |  |
| 1.645 |  |  |  |  |  |  |  |  |  |  |

**Fig5D**

|  | **TH2** | **TH4** | **TH5** | **TH9** | **SalI** |
| --- | --- | --- | --- | --- | --- |
| RC02 | 78.95 | 77.34 | 69.79 | 81.38 | 77.52 |
| RC03 | 86.60 | 85.22 | 49.85 | 48.97 | 50.39 |
| RC06 | 22.49 | 33.99 | 38.97 | 30.34 | 41.09 |
